# Effects of Residential Environmental Screening and Perception Surveys on Superfund Environmental Health Risk Perceptions

**DOI:** 10.3390/ijerph19138146

**Published:** 2022-07-02

**Authors:** Raja M. Nagisetty, William B. Macgregor, David Hutchins, Daniel A. Autenrieth, Alyssa M. Plant

**Affiliations:** 1 Department of Environmental Engineering, Montana Technological University, 1300 W. Park Street, Butte, MT 59701, USA; dhutchins@mtech.edu (D.H.); aplant@mtech.edu (A.M.P.); 2 Department of Professional and Technical Communications, Montana Technological University, 1300 W. Park Street, Butte, MT 59701, USA; bmacgregor@mtech.edu; 3 Department of Safety, Health and Industrial Hygiene, Montana Technological University, 1300 W. Park Street, Butte, MT 59701, USA; dautenrieth@mtech.edu

**Keywords:** superfund, environmental health perceptions, community engagement

## Abstract

Residents at one of the nation’s largest and longest-operating Superfund sites (Butte, Montana) have expressed environmental health risk perceptions that often diverge from those of EPA and other official stakeholders responsible for the investigation and remediation of site contamination aimed at protecting human health and the environment. A random sample of Butte residents participated in a study of how home-based environmental screening influences environmental health perceptions. Participants completed surveys measuring environmental health perceptions before and after a home site screening of soil and drinking water for lead and arsenic conducted by the research team. Local air monitoring for the same contaminants was also completed during the study period. The home-based screening intervention improved the alignment of subjective participant environmental health perceptions with objective environmental screening measures. Key features of the process that helped achieve this effect included (1) co-locating the collection of participant perceptions and individualized screening measurements; (2) sharing environmental screening results in a clear and unbiased manner; and (3) conducting this work independent of agencies and organizations with direct responsibility for Superfund-related cleanup activities. Empowering residents of a Superfund community with knowledge of the specific kinds and levels of environmental contamination in their home environment may help overcome the gap between agency conclusions regarding environmental health risk and the perceptions of community members.

## 1. Introduction

### 1.1. The Role of the Community in Superfund Decision-Making

In the more than forty years since it was established in law, the 1980 Comprehensive Environmental Response, Compensation, and Liability Act (CERCLA), better known as Superfund, has allocated billions of dollars to protect human health from environmental contamination caused by historic industrial activities [1]. Ten years earlier, in 1970, President Richard Nixon had created the United States Environmental Protection Agency (USEPA), and by 1980 with the passage of CERCLA, the Agency was given a mandate and a framework for overcoming environmental hazards anywhere in the country [2]. Superfund’s broadest foundational criterion by which it sets its cleanup goals is that the result must be “protective of human health and the environment”. Superfund’s Hazard Ranking system (HRS) is a numerical scoring system used by the U.S. Environmental Protection Agency (USEPA) to objectively evaluate relative threats to human health or the environment [3]. USEPA’s National Priorities List (NPL) as of 14 May 2021 [4] lists 1324 active sites.

The Agency’s highly structured approach to scoring the level of risk posed by contamination at a Superfund NPL site meets the program’s needs for scientific validation and confirmation of its determinations, providing objective and defensible measures to shape decision-making. However, another essential criterion for Superfund decision-making at any NPL site is “community acceptance”, which requires the Agency to do its best to ensure that the “affected community” has been involved in the process from the beginning and has a reasonable understanding of, and the opportunity to meaningfully contribute to, Agency decision making. Despite the USEPA’s recognition as to the importance of community engagement in Superfund communities, and substantial investments to improve communication and engagement with affected communities, an apparent disconnect between public perceptions of their environmental health and official environmental health assessments persists [5].

### 1.2. Community Environmental Health Perceptions

A previous study [5], developed a survey instrument to measure environmental health perceptions among Butte, MT residents who live in and around the largest Superfund site in the US. The study confirmed the existence of a perception disconnect between official reports of cleanup effectiveness at protecting human health and how residents personally perceive their own environmental health. Such a disconnect may undermine the community engagement process and may also exacerbate the suffering of communities in and around Superfund sites.

Understanding the role community perceptions play in efforts to improve community engagement has been the subject of numerous scholarly studies [6,7,8,9,10,11]. Such understanding is necessary to enable the public health agencies to achieve the goals and expected outcomes associated with community engagement, including, for many community members:

- Overcoming their disengagement from the community’s care for environmental health;
- Enhancing their ability to minimize their exposure to site contaminants; and
- Empowering them to participate meaningfully in influencing Superfund decision-making and its attendant implications for their personal health.

Research has confirmed that successful, sustainable site remediation requires giving as much attention to social implications of remedial plans as to the analyses of chemistry, geology, and biology at a site [12]. And even more pointedly, those social implications include considering how different demographic groups within a given community are likely to have significantly different perceptions about environmental health risk [13].

Nuanced and complex as they are, problems associated with community engagement have been the subject of many studies, and many of these were conducted specifically to develop comprehensive strategies to improve community engagement [14,15,16]. Elements of those strategies vary, but several relevant themes emerge:

- Ensure that residents have the knowledge required to understand health risks;
- Listen to residents’ pre-existing concerns about health risks and factor those into any interactions with them; and
- Treat residents’ “environment” as personal and local: where each of them lives.

To address these issues, recent research has explored ways that environmental health risk communication can be deployed to successfully engage local community members [17]. Recent development of health risk communication strategies has emerged as an active area of research [12]. This is the context of the current study’s focus and purpose.

### 1.3. Study Objectives

With the disconnect between official accounts of environmental health in the Butte, MT Superfund area and residents’ environmental health perceptions already established [5], the current study sought to explore if a simple environmental screening intervention could reduce the disconnect. The study’s overall goal was to measure what changes (if any) in residents’ environmental health perceptions would occur by providing residents with clear, accurate scientific information about Superfund-related environmental conditions in and around their homes.

The general approach involved measuring lead and arsenic concentrations in soil, water, and air in and around participants’ residences, while measuring their environmental health perceptions before-and-after sharing their environmental screening results with them. Thus, the study objectives were to evaluate the change in perceptions and the association between perceptions and environmental screening results. It was hypothesized that the disconnect would decrease and the association would increase following the intervention.

## 2. Methodology

### 2.1. Study Area

**Figure 1:**
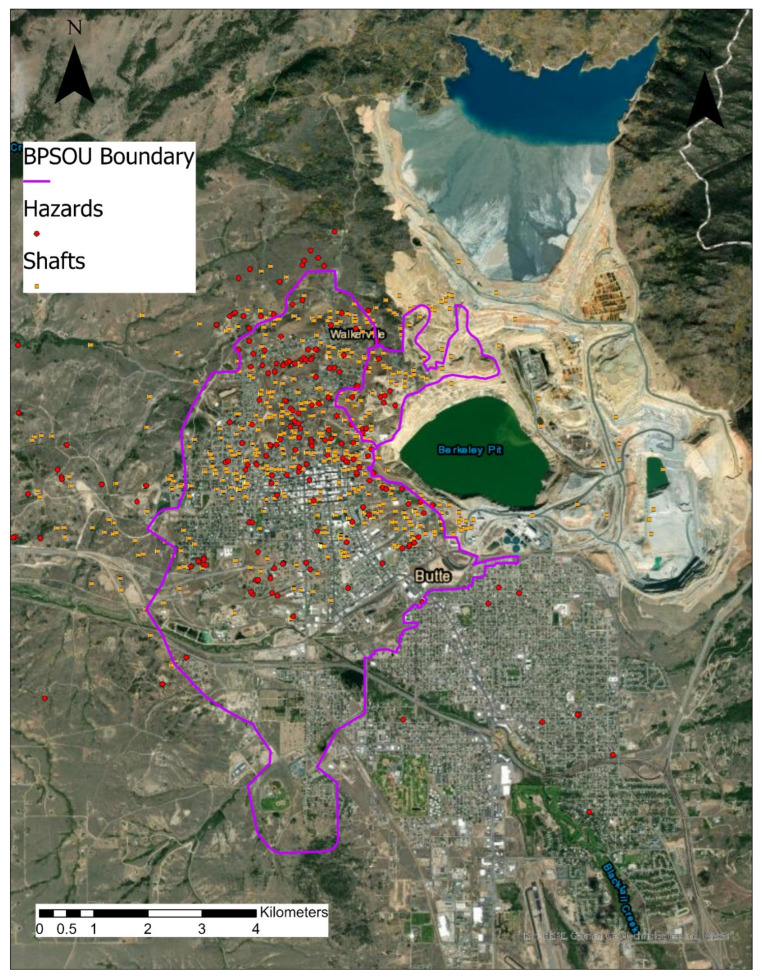
The overlay in this aerial perspective of the study area shows how intense and widespread historic mining activities were throughout the city, affecting virtually every neighborhood in the Uptown (north) area. “Shafts” marked in yellow indicate openings to vertical access to underground workings. “Hazards” marked in red reveal sites containing potentially hazardous mining wastes. Butte Priority Soils Operable Unit (BPSOU), Shafts and Hazards map layers were developed by Butte Silver Bow County and are available for public use. Authors have obtained these layers from Joseph Griffin (jgriffin2@mtech.edu).

Butte is a city of approximately 34,000 people situated near the continental divide in Southwest Montana (Figure 1) [18]. Established as a mining camp in 1864, Butte grew to be an industrial center of the region with the population peaking in the early 20th century. Under Superfund, the Silver Bow Creek/Butte Area was added to the National Priority List in 1983 [19]. The human health contaminants of concern (COCs) identified at this Superfund site include lead, arsenic, cadmium, and mercury (Pb, As, Cd, Hg). The primary exposure pathway is ingestion of solid media, namely soil and dust.

The 34,207 Butte Silver-bow residents are 49.5% female and 94% white [18]. The median household income is $42,174 with 66.7% living in owner-occupied housing. Census data show that 19% of the population lives in poverty.

Superfund sites are divided into Operable Units (OUs) based upon geographic areas of a site, specific site problems, or areas where a specific remedial action is required. The Butte Priority Soils Operable Unit (BPSOU) encompasses the residential portion of the city most heavily impacted by historic mine waste (Figure 1). The Residential Metals Abatement Program (RMAP) was established by EPA administrative order to test and remediate residential soils that have COCs that exceed an established action level.

The Mine Flooding Operable Unit (BMFOU) includes the underground mine works and the Berkeley Pit, which is a former open pit copper mine that currently contains over 150 billion liters of acid rock drainage water [20,21]. Currently, the level of the pit is maintained by active treatment and discharge into local surface waters. It should be noted that Butte drinking water sources are the Big Hole River, the Moulton Reservoir, and the Basin Creek Reservoir system, all of which come from outside the boundaries of the Superfund site and have not been impacted by mining waste [22,23].

Current mining takes place in and around the Continental Pit, adjacent to the city of Butte and historic mining. The open pit copper and molybdenum mine owned by Montana Resources Incorporated (MRI) is operated under strict modern regulation and monitoring under the oversight of Montana’s Department of Environmental Quality (MTDEQ) [24], and is not subject to enforcement actions under Superfund.

Previous environmental quality monitoring efforts have been extensive. The Remedial Investigation/Feasibility Study (RI/FS) phase of Superfund included studies of the soil, water, and air [25]. The studies found elevated levels of contaminants in soil, ground water, and surface water. The exposure pathways identified for residents were by ingestion of surface soils, interior dust, attic dust, and ground water. Current monitoring includes EPA assessments of soils outside of BPSOU, an expanded RMAP effort assessing residential soils and dusts, continuous monitoring of municipal water supplies, and multiple ambient air monitoring efforts [26,27,28].

Periodic medical monitoring is mandated by EPA administrative order to assess the effectiveness of remediation efforts [19,29]. These medical monitoring reviews are conducted every five years and largely focus on blood-lead levels, primarily among children. The first medical monitoring report was completed in 2014 and showed a sharp decline in historic blood-lead levels. However, the report identified slightly elevated blood-lead levels in Butte residents, as compared to national NHANES (National Health and Nutrition Examination Survey) data [30]. Additionally, it was noted that blood-lead levels in the BPSOU area were higher than they were among other Butte residents. The second medical monitoring report was published in 2020 [29]. Collection and analysis methods prevented rigorous comparisons to national data; however, local blood-lead levels continued to decline and appear to be plateauing near national levels. A study conducted by the Montana Cancer Surveillance and Epidemiology Program (MCSEP) noted that the cancer incidence rates in the Silver Bow County are comparable to those found among other residents of state of Montana and nationally [31,32]. In contrast, some studies have reported elevated exposure and health burdens in the community. One pilot study comparing blood arsenic (As) levels in a sample population of Butte with a comparable sample in Bozeman, Montana, noted that Butte levels are statistically elevated [33]. Another study utilizing death certificate records found that in the period from 2006 to 2016, cancers, cerebral and cardiovascular diseases, and organ failures appeared to be elevated in Butte-Silver Bow County as compared to other counties in Montana [34].

A protracted Superfund process, spanning over 35 years and including a federal gag order that prevented the principal parties from discussing cleanup plans with the public, has left the community exhausted and distrustful. Public engagement has been challenged by technocratic definitions of contamination, risk, and participation [35]. A previous pilot study [5], identified a disconnect between official agency health risk assessments, and residents’ actual mine-waste related environmental health perceptions. This disconnect was evident despite agency efforts at community engagement that have included public notices, public meetings, brochures and fact sheets, periodic interviews with community members, solicited feedback on proposed decision documents (and responses to that feedback). Likewise, official financial support has been provided for community groups that focus on Superfund issues like Citizens Technical Environmental Committee (CTEC, operating with EPA Technical Assistance Grant funding), Pitwatch.org (periodic tabloid newsletter about the Berkeley Pit), and cfwep.org (the Clark Fork Watershed Education Program) [36]. The good-faith efforts of the stakeholders in Butte’s Superfund operations to engage community members are clearly evident; yet the community perceptions of health risk persist.

### 2.2. Instrument Development

#### 2.2.1. Pre-Survey Instrument

To evaluate environmental health perceptions, a survey instrument was developed (Appendix A). The survey instrument consists of the following two sections: (1) potential environmental health concerns associated with this Superfund site, and (2) respondent demographic information. The Superfund-specific perception questions (Section 1) were followed by open-ended questions inquiring about experiences and information that may have shaped participant perceptions. Pre-intervention survey administration was co-located with the collection of environmental screening sampling in and around participants’ homes. For the Superfund perceptions questions, response was sought on a 6-point Likert–type scale (Appendix A), with response options of Very Good; Good; Somewhat Good; Somewhat Bad; Bad; Very Bad. For analysis, the responses were coded numerically ranging from 1 to 6, where 1 refers to very good and 6 refers to very bad. Respondents’ demographic information was sought in Section 2 of the pre-intervention survey, to assess whether responders are demographically representative of Butte residents’ demographics. Due to the small number of participants (*n* = 36) who completed the post-intervention survey, it was not possible to evaluate pre- vs. post-intervention perceptions change with respect to demographics. The survey instrument was an abridged and slightly modified version of a validated survey for this community developed in previously published work [5].

#### 2.2.2. Residential Environmental Screening Results Format

After environmental samples were analyzed for the presence of COCs, research team members prepared a document format for sharing the results of residential sampling of air, water, and soil with participants. The document contains the summarized background information about the results of the entire population being tested, but each resident’s form included data specific to their home situations, which no other participants saw. The goal was to provide sufficient technical information and context to both accurately convey the data gathered and clearly convey what the data suggest about levels of environmental health risk residents are faced with in their homes and yards. Afterward, participants, all of whom had already given their informed consent as described Section 2.3, were mailed the sampling results for their residences. The response format using a hypothetical example data is included in Appendix B.

#### 2.2.3. Post-Survey Instrument

The post-intervention survey instrument (Appendix C) was mailed to participants along with their environmental screening results. This post-intervention survey was identical to the pre-survey except for three minor differences, specifically, (a) this version didn’t ask for demographic information, which had been obtained previously, (b) this version added a new question about the effectiveness of the format of the environmental screening results document and (c) a new question was added asking participants their preference for receiving their incentive gift card.

### 2.3. Community Sampling and Subject Recruitment Approach

All subjects were recruited into the study using an Institutional Review Board (IRB) approved protocol. The population of the City of Butte resides primarily in one zip code: 59701. The HELPS lab at Montana State University [37], randomly selected 500 addresses that were stratified by age and income to oversample younger and lower income groups. This was done to address one of the limitations in the team’s previous perception assessment study [5], that is, the lower representation from low-income and younger age groups as compared to the community’s census data. Thus, these two groups were over-sampled to compensate for the expected lower-response rates in the current study. To recruit 50 randomly selected participants, the following inclusion criteria were used: (a) residents of Butte, Montana, (b) 18 years of age and above, (c) legally authorized to permit soil and water samples taken from their home and yard, and (d) any gender. The process emphasized ensuring participant representation from (a) RMAP-remediated houses, (b) the Butte Uptown area and (c) Butte Flats area in this list. Thus, as needed, additional potential addresses were chosen randomly from a list of homes that were remediated by RMAP and added to the mailing list. In total, 49 participants were recruited for the study, with five being from residences from RMAP remediated houses. While all the 49 participants completed pre-intervention survey, only 36 completed the environmental screening and post-intervention survey.

The subjects were informed of the research through a primer postcard notification sent in the mail. A week following the primer, a cover letter explaining the research was sent to the subjects. The cover letter explained the research objectives, participant role and incentive information and served as a consent document. To indicate interest in participating, respondents contacted the research team by phone or email. To encourage wider participation, the team offered all participants their choice of a $25 participation incentive in the form of a gift card. Consenting participants were contacted (either by phone or via email, per their preference) by the research group and provided with additional information about the survey and environmental sample collection process. The participants could fill out the survey questionnaire either in an electronic format or in a paper format, as they preferred. The electronic version of the survey was deployed using Qualtrics software and was identical to the paper survey. Direct contact between participants and the research team was minimized to comply with IRB requirements and to minimize virus transmission risk during the COVID-19 pandemic.

#### 2.3.1. Preliminary Perceptions Survey and Drinking Water and Yard Soil Sample Collection

After obtaining participants’ permissions, the research group left a sanitized sampling bottle and paper copy of the survey questionnaire (if the participant opted for a paper copy) outside the participant’s house. Participants filled the sampling bottle from their interior drinking water faucet and then left the bottle and the completed survey questionnaire outside their house. Researchers then picked up the sampling bottle. In the same manner, researchers collected the soil sample from the front and back yards at the residence, ensuring that residents maintained social distancing. Soil samples were collected for front and back yards for each household. A separate soil sample was collected from gardens at applicable homes.

#### 2.3.2. Air Sample Collection

**Figure 2:**
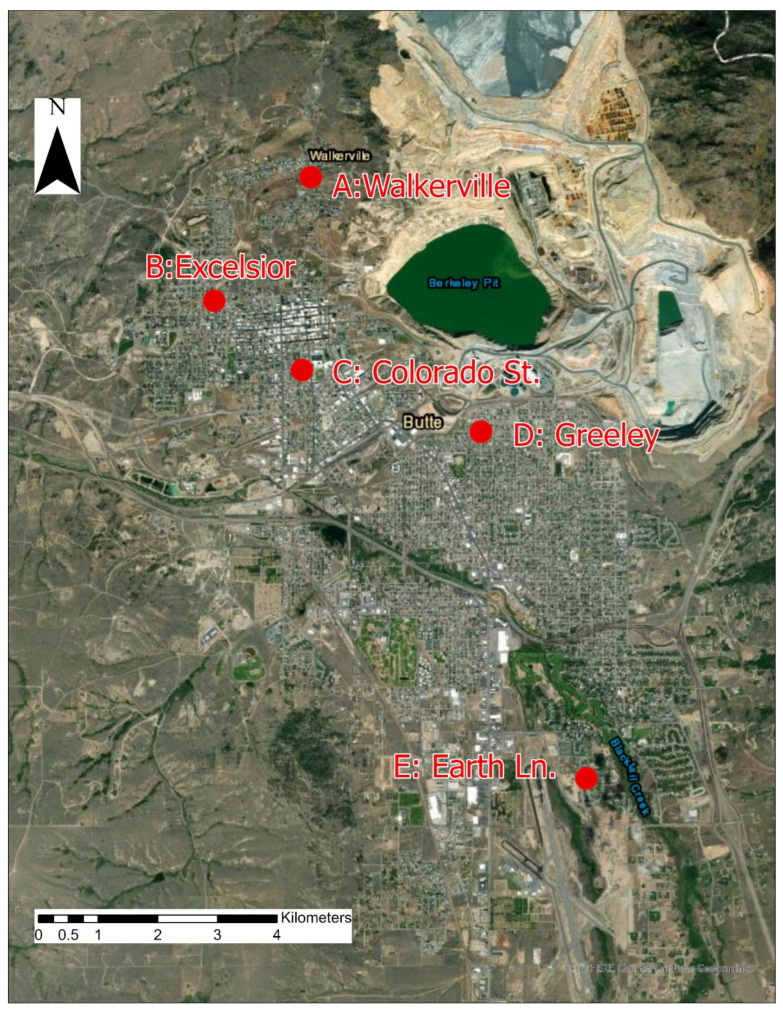
Study area map with air quality monitoring station locations.

Air monitoring for particulate matter (PM) was conducted at five locations throughout Butte over the same sampling period as residential soil and water (Figure 2). As previous research had indicated higher COC presence in larger aerosol size fractions [33], and other air monitoring results have found little-to-no COCs in the air when measured using total suspended particulate, coarse, and fine PM standards [27,38], an inhalable PM sampling method was employed. Each location utilized an Institute of Medicine (IOM) air sampler to collect the inhalable size fraction (50% cut point of 100 µm aerodynamic diameter) of ambient air PM (PM_100_). PM larger than 100 µm is collected on the filter with less than 50% efficiency while PM smaller than 100 µm is collected at a higher efficiency rate than 50%, and this size fraction captures all inhalable particles including the very small particles that reach the gas exchange region as well as the larger particles that deposit in the nasal passages and pharynx [39]. Inhalable size fraction was also of interest because lead and other contaminants of concern can be absorbed locally or through the gastrointestinal system when deposited in the upper regions of the respiratory system [40]. The air sampling station located nearest to the active mine had duplicate air samplers for quality control purposes, and the remaining sites had one each, for a total of six air stations at five locations. IOM air samplers were placed 5 feet above the ground under cover to measure PM concentrations at approximate breathing zone height. Air was sampled at 2 L/m for one week at a time with co-located field blanks present at the location nearest to the active mine, following the established IOM method [41]. Filters were changed every seven days for six consecutive weeks, totaling 42 filters available for analysis. No filters were overloaded during the study period. Particulate was collected on 25 mm PVC filters that were desiccated and pre-weighed before use. After sampling, each filter was dried and post-weighed for gravimetric analysis using a calibrated, precision microbalance.

#### 2.3.3. Post-Perception Data Report and Analysis

The mailing packet containing the sampling results included a second cover letter and the post-intervention survey. The packet also contained a copy of the “Be Contaminant Smart brochure” prepared by CTEC and EPA. Participants were asked to review their environmental screening results and contact information was provided for a research team member to answer any follow up questions they might have. Participants were asked to fill out and return the post-intervention survey after they had reviewed their results and discussed them further with the research team, as necessary.

### 2.4. Laboratory Analysis

All the water, soil and air samples were analyzed for elements of interest by a certified third-party laboratory (Energy Laboratory, www.energylab.com, accessed on 30 June 2022). Drinking water samples were analyzed using the E200.2 (total metals digestion) and E200.8 method for Contaminants of Concern (COCs; As and Pb). Soil samples were analyzed using E6010.2 method for As and E6010.20 method for Pb. Dust Samples were digested, diluted, filtered and analyzed using ICP-MS for the presence of Pb, As, in addition to copper (Cu), manganese (Mn), molybdenum (Mo), and zinc (Zn) according to the EPA method 6020A protocol. It should be noted that for Butte Superfund site COCs in dust are As and Pb. For this study, Cu, Mn, Mo, and Zn were analyzed as additional elements of interest to the community.

### 2.5. Statistical Data Analysis

The effectiveness of the intervention was evaluated by analyzing pre- and post-intervention perception responses using interval plots (for changes) and odds ratios (for associations). Microsoft Excel 2019 version 1808 (Redmond, WA, USA) and Minitab Statistical Software version 19.2020.1 (State College, PA, USA) were used to organize and analyze the data.

#### 2.5.1. Change in Participant Perceptions

Interval plots were developed to compare subjects’ mean environmental health perceptions before and after learning about the concentrations of COCs in and around their homes for the four-exposure media (drinking water, air, yard soil, and garden soil). The differences between paired pre- and post-survey responses were analyzed using 1-Sample Sign Test (2-sided, α = 0.05) with a hypothesized difference of zero. The null hypothesis was that there was no difference in the paired means for each exposure medium.

#### 2.5.2. Odds Ratio Analysis

Odds ratios analysis was performed between the yard soil COC (As and Pb) concentration category of high versus low (above or below the EPA or MT DEQ action level) and soil perception category, which were positive (ratings of 1–3) or negative (ratings of 4–6) as reported by subjects before and after receiving their yard soil screening results (*n* = 35). Chi-squared test of independence (2 × 2 contingency table; α = 0.05) was used to test the effect size of the association. The null hypothesis is that there is no association between binary concentration category and perception categories. We did not perform Odds ratio analysis for drinking water, air, and garden, as there were no EPA action level exceedances for these exposure media.

## 3. Results

### 3.1. Co-Located Residential Environmental Quality and Health Perceptions

The co-located data were collected for two purposes: (a) to provide contemporaneous data for preparing informational material and strategies relevant to individual residents, and (b) to identify any connections that may exist between the extent of COCs exceedances and associated perceptions of environmental health risk among residents. The following sections discuss the results of the co-located data collection efforts in terms of the three-exposure media: water, air and soil (including gardening).

#### 3.1.1. Water

**Figure 3:**
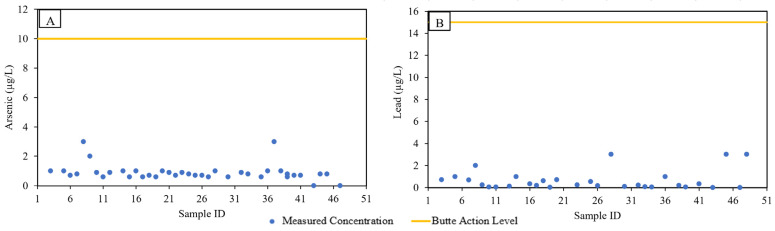
Butte residential drinking water As and Pb concentrations. (**A**) Drinking water As concentrations; (**B**) Drinking water Pb concentrations.

Butte drinking water (a) is sourced outside of mining-impacted surface water bodies, (b) is treated, and (c) complies with mandatory federal standards [22,23]. The USEPA Maximum Concentration Level (MCL) for As and Action Level (AL) for Pb are 10 and 15 µg/L, respectively [42]. As expected, none of the residential drinking water samples (*n* = 48) exceeded MCL or AL for either As or Pb (Figure 3). For most of the samples, the concentrations were an order of magnitude lower than the MCL or AL. Butte-Silver Bow Water Utility Division monitors drinking water in accordance with Federal and State laws and regulations. Monitoring reports are published online and available to public for review. The BSB Water Division’s 2020 annual drinking water quality report shows that As concentrations were always non-detect and for Pb, the highest concentration measured (at 90th percentile) was 12.67 µg/L (for Basin Creek treatment plant), and that concentrations never exceeded the action level of 15 µg/L [28].

With respect to the drinking water, the information provided in the “individualized residential screening results” document include—(a) the range of values measured in this study, (b) USEPA action levels, and (c) the corresponding participant residence concentrations. This data informed the residents about the status of their residential drinking water. Based on the literature review and data collected in this study, the research team’s reports to residents showed that Butte drinking water coming into their homes meets Federal and State regulations for safety and health.

#### 3.1.2. Air

Of the five monitoring stations, detectable concentrations for Cu, Mo and Zn were measured only at two stations—C and D (Figure 2, Table 1). These trace metals appeared in barely detectable quantities. The other three elements (Pb, Mn and As) were not detected at these two stations. At the three remaining monitoring sites (A, B, and E, Figure 2) none of the elements of interest were detected (Table 1). Please note that a duplicate monitoring station was placed at location D (Table 1) in anticipation for a number of low concentrations and non-detects in the air monitoring data based on previous studies using different aerosol size fractions [27,33,38]. Our quality assurance goal was to ensure that at least 80% of the measurements were in agreement with regard to detect versus non-detect. We met this quality assurance goal (Table 1). Standards or action levels for these elements of interest are not well established. However, the levels observed in this study do not raise immediate health concerns. None of the measurements made during the six-week sampling period showed evidence of airborne lead concentrations that exceed the established national standards of air quality [43].

These results are consistent with contemporary published data from other sources, which found little-to-no evidence of COC exposure in air, as measured using TSP, PM10, and PM2.5 standards [27,33,38].

For air quality, the information provided to residents in the “individualized residential screening results” document included—(a) a map showing five sampling locations, and (b) write-up of results. The data collected as part of this study and contemporary literature clearly shows no exceedance for elements of concern.

#### 3.1.3. Soil

**Figure 4:**
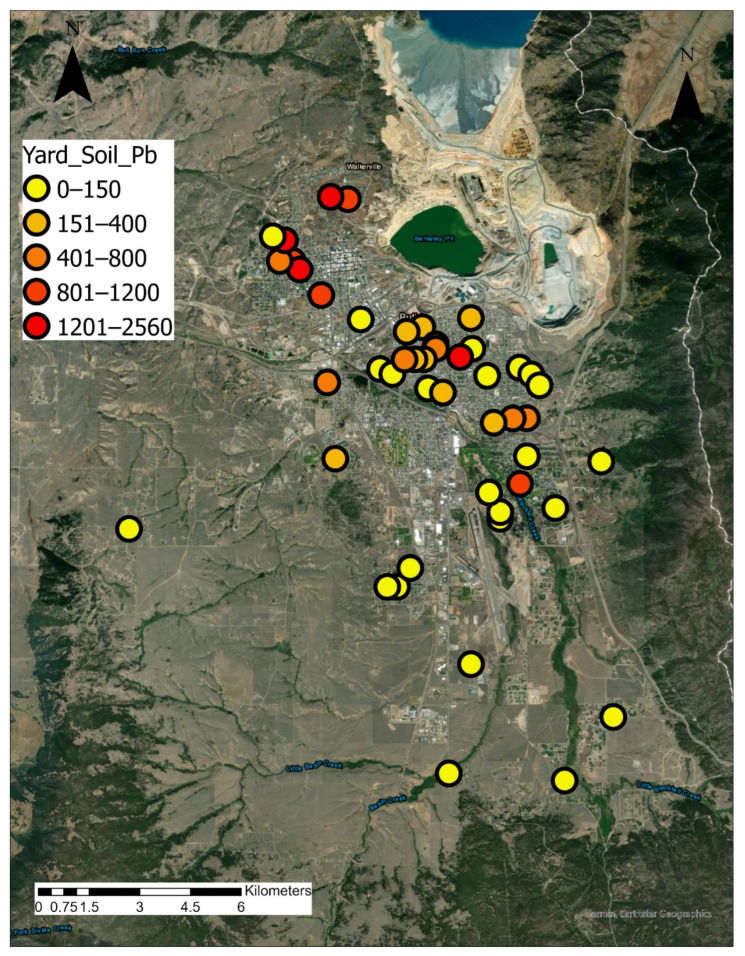
Aerial view of Butte showing Pb concentrations at each residence sampled. Note that distribution of sites throughout the city, both in the midst of historic mining activities and farther away. Levels of Pb shown represent the maximum value of front yard, back yard, and garden soil at each site.

Figure 4 presents an aerial view of Butte with Pb concentrations at each residence sampled. Levels of Pb shown represent the maximum value of front yard, back yard, and garden soil at each residence sampled. As discussed in Section 2.1, historic mine waste deposition has resulted in widespread contamination of yard soil. As part of programmatic remediation efforts, the RMAP program has been introduced to remediate residences, if As, Pb or Hg concentrations exceed Butte site-specific action levels. USEPA Butte site-specific action levels for As and Pb in residential soils are 250 and 1200 mg/kg, respectively [25]. The RMAP program has been effective with 1227 completed abatement projects and has sampled 3189 residential parcels as of 31 December 2017 [44]. USEPA national default action levels Pb in residential areas is 400 ppm [45]. Montana’s Department of Environmental Quality action level for As in residential soils is 40 ppm [33,46]. It is also important to note that within the same superfund site, for a nearby town (Anaconda Smelter Site Community Soil Operable Unit), the action level for Pb is 400 ppm [47]. The national default levels are used when no site-specific data is available that might indicate a different standard of protectiveness. In Butte, studies have shown that the action levels can be adjusted higher because of reduced bioavailability. Because the Pb and As found in Butte is typically in a mineral matrix, it has been reported to be less bioavailable than elsewhere [48]. This difference in action levels has been a source of increased public concern in Butte about the protectiveness of Butte’s action levels, and thus may affect residents’ perceptions about the results of soil screening at their residences.

**Figure 5:**
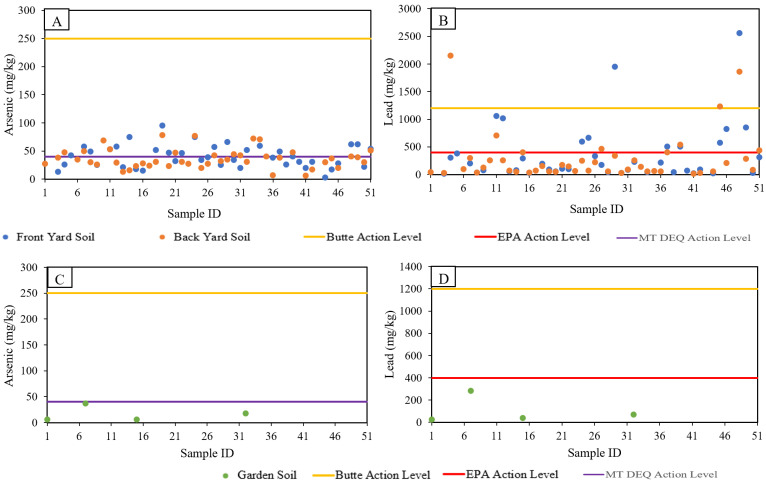
Butte area residential yard soil and garden soil As and Pb concentrations. (**A**) Residential soil As concentrations; (**B**) Residential soil Pb concentrations; (**C**) Garden As concentrations; (**D**) Garden Pb concentrations.

None of the residential samples taken in this study (*n* = 48) exceeded site-specific USEPA action levels for As (Figure 5). However, 33.3% of samples exceeded the MT DEQ action level. For Pb, 8.3% of samples exceeded site-specific action levels and 31.3% of samples exceeded the national USEPA action level (Figure 4 and Figure 5). For soil, the information provided in the “individualized residential screening results” document included, (a) agency site-specific action levels, (b) agency national action levels, (c) explanation of difference between national and site-specific action levels, (d) range of concentrations measured in this study, and (e) individual resident yard soil concentrations. The write-up also mentions that “in fact there is no safe level of lead exposure and you should always eliminate any exposure you can”. Unlike water and air, the soil quality presentation called for a more nuanced explanation of its significance.

### 3.2. Effects of Residential Environmental Quality Data Material Sharing

The effects of residential screening results on residents’ perceptions varied with each exposure medium; the following sections discuss how those effects were manifested in residents’ post-survey ratings and commentaries.

#### 3.2.1. Water

**Figure 6:**
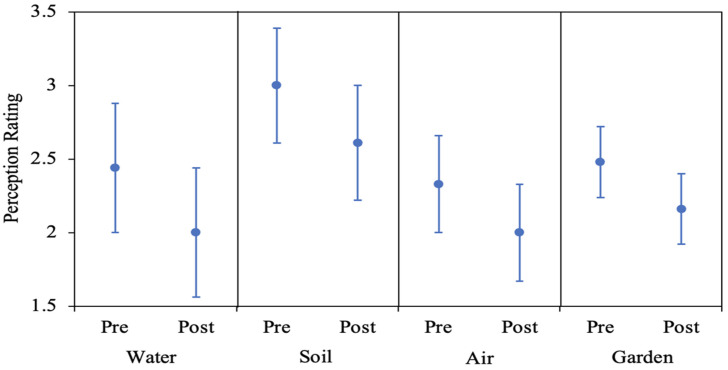
Interval plots comparing mean and 95% confidence intervals perception ratings before and after intervention for the three-exposure media (water, air, soil, plus garden). Y-axis title ‘perception rating’ refers to participants perception response on a 6-point Likert-type Scale: 1: Very Good; 2: Good; 3: Somewhat Good; 4: Somewhat Bad; 5: Bad; 6: Very Bad.

**Figure 7:**
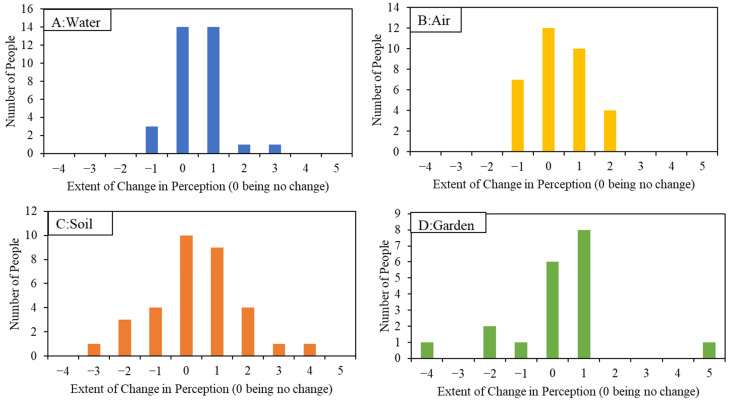
Aggregated change in perceptions before and after intervention. Change in perception is the perception difference between before and after intervention rating. Perceptions were reported on a 6-point Likert-type Scale: 1: Very Good; 2: Good; 3: Somewhat Good; 4: Somewhat Bad; 5: Bad; 6: Very Bad. So, Positive x-axis numbers (meaning perception changed from a higher number to a lower number) indicate perceptions have improved.

The average perception changed from 2.44 from pre-intervention to 2.00, post-intervention (Figure 6). The reduction (improvement) was statistically significant (*p* = 0.007). Analysis of change in perceptions pre- and post-intervention indicated that the majority of change was towards positive perceptions (Figure 7). Pre-intervention, 20.8% responders reported negative perceptions for drinking water (i.e., selected either very bad, bad, or somewhat bad). Post-intervention, only 13.9% reported negative perceptions. It should be noted that the residential screen results material suggests that drinking water quality is very good, quality is monitored, and no concerns were expressed. It appears that the message has informed perceptions to a certain extent (Figure 7). This conclusion can also be supported by reviewing the qualitative comments provided by the participants. Pre-intervention, taste (*n* = 7), location (*n* = 7), treatment system (*n* = 5) were the top three reasons mentioned for the perceptions. Post-data sharing, nine participants indicated that their beliefs were validated, and six participants expressed relief to know that their Pb and As levels were low.

#### 3.2.2. Air

For air quality specifically, the mean perception changed from 2.33 before intervention to 2.00 after intervention (Figure 6), and this improvement was not statistically significant (*p* = 0.307). Pre-intervention, 14.29% responders reported negative perceptions as compared to only 2.78% indicating negative perceptions post-intervention. Analysis of change in perceptions before and after intervention indicates that the majority of change is towards positive perceptions (Figure 6). However, unlike drinking water, seven participants have decreased their perception rating (Figure 7). The only common theme in the qualitative responses pre- or post-intervention was that the air quality data was not collected at or in participants home (*n* = 5).

#### 3.2.3. Soil

The mean perception for yard soil changed from 3.00 before intervention to 2.61 after intervention (Figure 6), and this improvement was not statistically significant (*p* = 0.248). For yard soil quality, 38.8% of participants indicated negative perceptions pre-intervention, while 22.2% indicated a negative perception post-intervention. The major themes residents used to explain their positive or negative response to the data were: low levels (*n* = 9), levels higher than expected, and levels seem high (*n* = 7). The contradicting themes were not surprising due to the variation in yard soil COCs concentrations. Some participants’ yard soil concentrations were low and some participants’ concentrations were elevated. This contradiction is also reflected in how perceptions changed with residential screening results sharing (Figure 7). Unlike water or air, the change in perceptions was distributed both on positive and negative sides (Figure 7). As expected, positive and negative perceptions seemed to be informed by the residential screening results.

The mean perception for garden soil changed from 2.48 to 2.16 after intervention (Figure 6). Fewer responses in this category reflect the fact that most participants did not have a garden. For garden quality, 23.5% of participants who responded indicated negative perceptions. Post-intervention, 12% of participants indicated negative perceptions, but this improvement was not statistically significant (*p* = 0.180). The major themes in the post-data share responses were brought in soil (*n* = 4), and the results have raised confidence (*n* = 3). Perceptions changed in both directions: positive and negative (Figure 7).

#### 3.2.4. Three-Way Instead of Binary Distribution of Perception Changes

**Figure 8:**
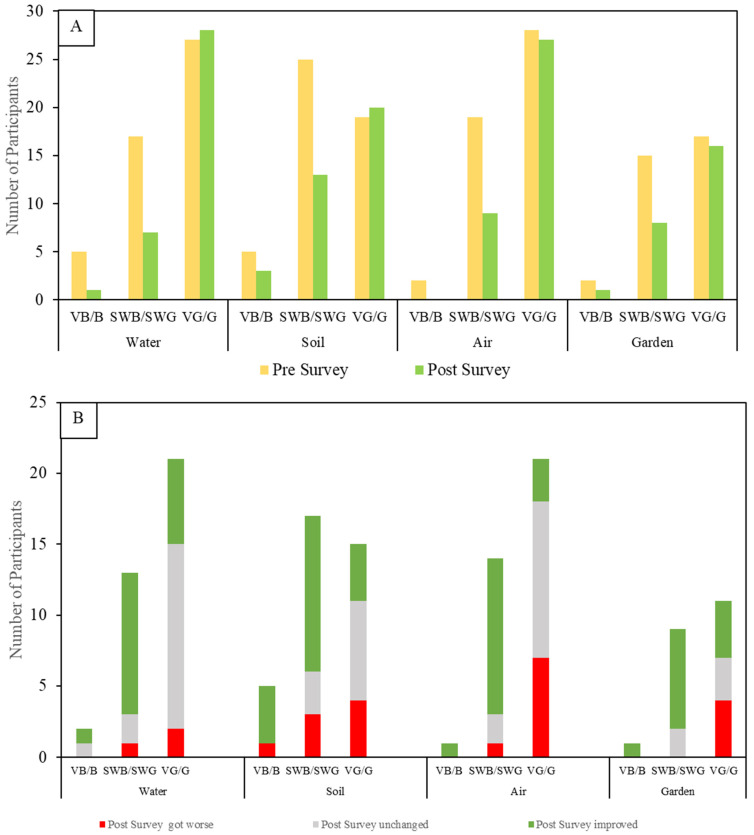
Three-way perception analysis. (**A**) tracking changes in perceptions of environmental health concerns before and after intervention. (**B**) rating change analysis (positive, remained same or negative). It should be noted that post-intervention, only 73% of participants participated. VB/B: very bad or bad; SWB/SWG: somewhat bad or somewhat good, VG/G: very good or good.

Another way of assessing the effects on residents’ perceptions is to look at changes not merely as positive or negative, but to separate responses in the good and bad extremes alongside those in the middle: that is, how did people whose initial perceptions were rated very bad or bad, for instance, change once they received the results of environmental screening in and around their homes? Did the information they received make their perceptions become more positive, more negative, or stay the same? Figure 8A shows the changes from pre- to post-intervention.

By separating out the somewhat bad and somewhat good middle ratings from the two extremes, we draw attention to a likely tendency for some respondents in the beginning to avoid expressing very positive or very negative views. Such response patterns are variously known in psychometric literature as central tendency bias, or end-aversion bias [49]. This pattern is evident in both charts in Figure 8: the “somewhats” in the middle were dominant during the pre-survey, but in their responses to the post-survey, residents moved decisively away from the middle and toward the positive, suggesting that sharing the screening results gave them the confidence to make a more definitive judgment. For example, 51% of participants selected “somewhats” before seeing the soil data and only 36% selected “somewhats” after seeing the data. A similar shift appears for water and air, in roughly the same frequency, and the pattern corresponds to an even greater increase in post-survey responses for all media in the VG/G (very good/good) category. Also, it is reassuring that for all media, the post-survey ratings of VB/B (very bad/bad) were reduced to three or fewer respondents, suggesting that sharing personalized information may have curtailed the most negative extremes of perceptions (Figure 8A).

To explore the changes in initial perceptions after sharing environmental information about their homes, the percentages of respondents whose ratings improved, stayed the same, and got worse gives some insight into the ways the information received affected residents’ perceptions (Figure 8B). Several insights emerge from Figure 8B: most of the instances of resident ratings getting more negative by a point or more after seeing their screening results show up in the most positive initial raters: that is, the screening results appeared to sharpen the focus of many of those who initially scored their water, soil, or air as very good or good. In most of these cases, “getting worse” meant ratings that moved only from very good to good, and that may be suggestive that sharing the results made those who were most positive to begin with more sensitive to the implications of the comparative data they received. Figure 8B also shows that, except for soil, all respondents rating the media as very bad or bad had their perceptions either improve or stay unchanged after receiving the screening information.

### 3.3. Odds Ratio Analysis Results: Correlation between Perception Changes and Screening Results

**Figure 9:**
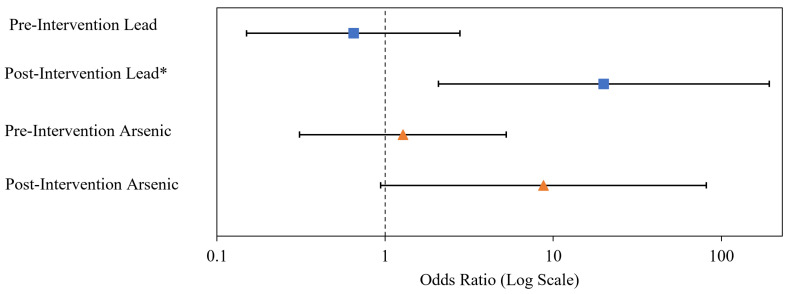
Odds ratios between the soil concentration category of arsenic and lead (above or below the EPA action level) and soil quality perception category (negative or positive) as reported by subjects before and after receiving information on the metal concentrations in their yard (*n* = 35). * The association was significant at the alpha = 0.05 level.

An Odds Ratio Analysis (Section 2.5.2) aimed to assess the association between binary environmental health perception (good vs. bad) and binary environmental screening results (high vs. low), and to determine if the apparent relationship changed post-intervention (Figure 9). For the purpose of the odds ratio analysis, (a) for Pb, an “elevated” concentration was defined as concentrations greater than EPA national action level; (a) for As, an “elevated” concentration was defined as concentration greater than the MT DEQ action level of 40 mg/Kg for all parts of Montana. The researchers used this alternative definition, because the number of exceedances of EPA’s Butte site specific action level were minimal. No significant association was evident for Pb or As, pre-intervention (X^2^ of 0.34 and 0.12 with *p* = 0.561 and 0.730 for Pb and As, respectively). The apparent association increased post-intervention for both contaminants, and the association became significant for Pb (X^2^ of 9.75 and *p* = 0.002), but the association for A_S_ was not quite significant (X^2^ of 4.61 and *p* = 0.082). We were not able to perform similar odds ratio analysis for drinking water, air, and garden soil, as there were no EPA action level exceedances for these exposure media.

### 3.4. Categorizing Participant Comments

Participant comments resolved into six general categories, revealing the variety and frequency of different perceptions & responses regarding water, soil, and air quality. Table 2 shows the number of instances of participant comments relating to each category for each medium sampled, identifying patterns of perceptions that effectively characterize the perceptual environment in which the intervention took place.

For instance, participants reported strong familiarity with, and trust in, the widely understood improvements made to the municipal water system under Superfund, giving them confidence in the quality of water in their homes. The odds ratio analysis for Pb shown in Figure 8 may help explain why 44.5% of post-survey responses about drinking water remained unchanged, possibly reflecting the alignment of perceptions with the site-specific data.

In the case of soil, the widely known and used RMAP program that samples and removes contaminated residential soil and dust yielded the highest percentage of responses, suggesting initial knowledge of, and trust in, these official policies and procedures. Frequent emphases among commenters on proximity to historic mining sites, and the appearance, texture, and character of the home’s soil (Table 2) largely correlated with the variations in soil-Pb screening data that were shared with the residents. This suggests that these kinds of the most readily perceived evidence may predispose residents to link their personal experiences with environmental data collected at the site.

By itself the correlation of initial trust in the system and the science governing remedial decisions and activities alongside the participants’ sense of having their perceptions validated suggests that any existing perception gap has the potential to be overcome by reinforcing a climate of trust with personalized data collection and sharing activities as demonstrated in this study.

Reasons given for changes reported in levels of concern, both as initially recorded, and as reported after residential screening results vary among water, soil, and air. For drinking water comments (*n* = 74, representing the tally of multiple comments from participating residents), the largest group of respondents (*n* = 20) expressed satisfaction that the screening results confirmed what they already believed. The second largest group (*n* = 16) of respondents mentioned the official entities responsible for ensuring the safety of the water. Tied for third (*n* = 10–11) were those paying heed to location (proximity to mining wastes), the smell, taste, and appearance of the water, and their prior experiences with Butte’s drinking water.

For soil comments (*n* = 87) the largest group (*n* = 24) drew attention to the soil’s appearance, in many cases contrasting it with “normal garden-type soil”; in fact, respondent comments about garden soil were almost as frequent under this category as they were under the specific set of questions about gardening. The two second largest groups (*n* = 18) included observations about location, not surprisingly focusing on the proximity of the residence to historic mining wastes and uptown home sites, as well as registering satisfaction that their existing perceptions were confirmed by the screening results. Finally, a dozen respondents expressed their perceptions about potential contamination of soils in the context of official oversight, with most saying something to the effect that “if something had been wrong, they would have told me”.

Comments related to air quality were less frequent (*n* = 64). The largest category of comments about air (*n* = 19) conveyed the sense that the results they received confirmed their initial perceptions and beliefs. The only other category with more than 10 comments alluded to their prior experiences of air quality, either positive or negative (*n* = 13).

## 4. Discussion

### 4.1. Complex Effects of Informing Community Members about the Presence of COCs in and around Their Homes

The study’s pre- and post-quantitative survey results need to be understood in the context of the actual measured and reported sampling results. It doesn’t make sense to assume that more information should always make people feel better, that is, improve their perceptions, about their situation: if they initially thought their yard soil was safe and their water was clean, but sample results showed higher than expected levels of Pb in the soil, the new information should lead them to be more concerned than they were initially—and their post-survey responses should reflect that increased concern with a worse rating than their first one. On the other hand, when fearful residents rated their water quality or soil cleanliness as bad or very bad, and the sample results showed the opposite, one would expect to see ratings improve dramatically, which in many instances, is exactly what we found (Figure 7 and Figure 8). In both cases, changes in perception after sampling should correlate to what was found on site: it’s that correlation that indicates the success of information sharing the study was looking for (Figure 8).

One earlier study that explores many of the same issues involved with responding to community health risk perceptions associated with exposure to contaminants from a mining operation [6], offers applicable insights, as well as contrasts to the current study of Superfund activities at the Silver Bow Creek/Butte Area NPL site in Montana. Catalan-Vazquez et al. [6], interviewed over 400 people living in multiple neighboring communities affected by the manganese mining operations in the Molango mining district in Mexico. The purpose was to acquire a detailed account of the range of perceptions about health risks throughout the population across multiple communities, and to try to explain the patterns that emerged from the data collected. That process and purpose is shared by the current study of Butte’s Superfund site.

What differentiates this study from the Catalan-Vazquez et al. [6], study is that the Butte study was built on the prior establishment of a disconnect between officially sanctioned, scientifically determined assessments of health risk perceptions and residents’ actual mine-waste related environmental health concerns regarding the presence of officially listed COCs (Contaminants of Concern—in this case, lead, arsenic, and mercury) in the exposure media (drinking water, air and soil) at the Superfund site where they live—that is, anywhere in the affected community [5]. The current study specifically aimed to assess how the perception disconnect or gap is affected by examining the actual exposure data onsite in people’s homes and communicating that data to residents—the assumption being that the perception disconnect may be a simple function of a lack of relevant information and that supplying such immediately applicable information will help overcome that disconnect. Unlike the Catalan-Vazquez et al. [6], study, the Butte study moved beyond analyzing possible reasons for health risk perceptions among the affected population, to implementing a process of engagement aimed at more effectively informing that population to help overcome the perception gap.

In the initial surveys some of the commenters often expressed confidence in the environmental health of the water, soil, and air around their homes. But even those participants sometimes expressed skepticism about the environment of the town in general, as evidenced by the large percentage of comments that explained their lack of concern by noting their residence’s lack of proximity to the “bad (contaminated) part of town” (Table 2). This confirms the findings of Catalan-Vazquez, et al. [6], which revealed a behavioral pattern in which residents perceptually displaced environmental concerns away from their home neighborhood environment and projected those concerns out onto the community at large, a phenomenon known as the “neighborhood halo effect” [6,50]. In the current study, the range of responses to the screening results varied from expressions of gratitude and appreciation for the screening and the sharing of information, to expressions of distrust and skepticism.

One of the patterns that emerges from both the quantitative (Likert ratings; Figure 7, Figure 8 and Figure 9) and qualitative (survey comments; Table 2) aspects of this study largely relates to the issue of trust. Taken together, the data collected suggest that environmental sampling that is done, as this was, in a public health environment, as opposed to a regulatory enforcement environment (i.e., conducted by government entities charged with enforcing environmental laws), can in fact be an effective way of “bridging the gap” between public perceptions about health risks posed by localized COCs and official pronouncements about such risks. The study’s individualized treatment of participating residents, by an independent and trusted entity only interested in informing the residents about the sampled presence of COCs, an entity not empowered to force them or anyone else to do anything about the findings, appears in the responses of survey participants to have largely overcome a pervasive atmosphere of mistrust of officially sanctioned findings of environmental health risk.

The predominance of post-survey responses expressing satisfaction that their initial perceptions about the environmental health in their homes were largely confirmed by the results reported from the screening process suggest that one major benefit of the process is to build trust among a population who have been told for more than 30 years that their homes exist in a contaminated environment, a Superfund site (Table 2). The relief is palpable among many of the comments received. Even in cases where higher levels of contaminants were reported than residents expected, most residents’ comments suggest that they are reacting as they should to this new information: with a new level of caution and alertness to potential environmental hazards, which is exactly the perception such information should lead them to.

On the other hand, a significant, though small, percentage of respondents whose post-survey responses reflect uncertainty and/or a rejection of the results of the screening process suggest that pre-existing attitudes, experiences, and perceptions among this group prevent them from readily accepting information that conflicts with those personal attitudes and experiences. For these people, the straightforward act of providing clear, relevant, and personally applicable scientific information appears not to be enough to overcome those prior perceptions. In these cases, deeper engagement is needed if the perception gap is to be bridged. This presents a clear opportunity and need for future study.

### 4.2. Balancing Insights from Binary and Three-Way Scalar Analyses

Whether response data is analyzed on a binary (bad/good) scale, or a three-way scale (two extremes vs. middle), suggestive patterns emerge that provide insights into participants’ concerns. This supports the central purpose of the study, which was to test the hypothesis that providing residents with clearly presented information about potential environmental contaminants in their home environments can change their perceptions to correspond with the information presented. While the study’s relatively small post-survey sample size limits the study’s ability to draw definitive statistically defensible conclusions, the wealth of insights gained from the qualitative data contained in the comments clearly shows changes in perceptions following the data sharing as discussed in Section 3.4. The patterns of concerns that emerge from these comments show a range of points of contact that such communication efforts must consider.

Both of our two analyses of perception measures and changes in those perceptions tend to confirm our primary hypothesis: that informing residents about actual environmental conditions in and around their homes will help connect them to the perspectives of the official entities charged with protecting public health. The primary distinction between the two approaches is that the three-way analysis may be interpreted to show that the respondents reporting an initial slightly positive or slightly negative perception of risk may not be well-enough informed to make a more decisive judgment. The post-intervention movement of these perceptions from the middle toward a more positive direction suggests the potential value inherent in conducting this sort of risk communication and data intervention activity with members of the community. The strength of the binary approach lies in the suggestiveness of its broader measures, in which the responses provide a simpler sense of affirmation of the effectiveness of this approach to risk communication.

### 4.3. Extrapolating Insights to Other Superfund Sites

Both quantitative and qualitative perceptions data need to be collected at regular intervals to identify, quantify, and characterize the perception gap at regular intervals and develop community engagement material accordingly. While quantitative data (e.g., Likert scale) help scale the extent of certain perceptions, qualitative data provide understanding of the extent and source of certain perceptions. Combining both types of data sets (as performed in this study) provides insights to developing material that can more effectively inform the perceptions. The major difference between the previous study [5], and the current study is that this study focuses on personalization: the perceptions of environmental exposures in their residential setting. Perception data collected becomes more focused and actionable when the questions are specific and personalized. The study results suggest that residents in a Superfund community highly appreciate such individualized and independent third-party data collection, analysis, and presentation efforts.

For legacy Superfund sites, one of the challenges in perception data collection is separating perceptions resulting from historic experiences to current conditions. The perceptions data collected in this study are designed to capture a snapshot of current perceptions at a specific place and time. However, from the qualitative data it can be seen that some of the participants’ perceptions emerge from their historic experiences, making it difficult to filter out those long-standing perceptions to empower their ability to recognize and accept currently relevant data.

One of the end goals of community engagement at Superfund sites is to make community members aware of their environmental health risks and strategies to actively mitigate potential exposures (if any). In addition to their residential environmental screening results, the researchers also shared contact information for the local remediation program, as well as a booklet titled “Be Contaminant Smart” prepared by CTEC and EPA. Among other material, the booklet presents strategies to minimize exposure to COCs. While the researchers neither prepared this booklet nor sought participants’ feedback on it, this document very likely helped shape residents’ post-survey perceptions. The study, while conducted at a site that’s been a legacy superfund site, offers insights for new or relatively younger superfund sites. Future investigations should include developing guidance for preparing community engagement plans to preemptively minimize the potential for building up the kind of perception gap experienced in Butte.

### 4.4. Limitations

One of the limitations of this study is its participant sample size. Initially, the research group recruited 49 participants (randomly selected) for the study. These participants completed pre-intervention questionnaire and participated in environmental sampling. However, only 36 participants responded post- intervention (73% response rate). While all the participants are randomly selected, the smaller sampling size makes the process of drawing inferences about the changes in residents’ perceptions at best suggestive, but not decisive. Moreover, the study was performed during COVID-19 disruption, and this affected not only the study’s methodology, but it is possible that the on-going pandemic might have influenced participants’ perceptions. One of the study’s missed opportunities was a deeper investigation of what difference the format, style, and approach to information-sharing can make in community understanding and acceptance of environmental health data; we included a brief question about the effectiveness of the information-sharing materials presented to them, but we didn’t receive any insights from the feedback to that question. This leaves ambiguous the degree to which the way the information was presented might have affected residents’ post-screening surveys. A separate part of this investigation undertook to address this concern and is currently finalizing its results. Finally, some of the results reported regarding perceptions about water quality (such as taste and appearance) may well have been influenced by the fact that at least two residents were describing their home’s well-water supply, not city water.

## 5. Conclusions

The study concludes that some of the perception gap noted in our previous study (Nagisetty et al., 2020) can be bridged by sharing contemporary, individual, and contextualized contaminant exposure data with the affected population. Prior to this sharing of information in this study, some of the participants were aware of the exposure risks while some of them were not. Additionally, some of the participants based their risk perception on knowledge of environmental quality and some of them based it upon erroneous assumptions. In a significant number of cases, exposure to individualized and contextualized data led to a more informed perception of risk, more closely aligned with scientific understandings of contaminant exposure, and empowering residents with a clearer understanding of environmental issues they may or may not need to attend to around their homes. Interval plots and Odds ratio analysis suggest that the apparent association, between perceptions and residential environmental quality, increased post-intervention.

These findings have potential application beyond the study area. To bridge the gap at other Superfund sites, the current study has shown that an individualized data-centered approach to community engagement can be effective. The study also suggests that the residents appreciate independent third-party data collection, analysis, and presentation efforts. Community members that feel heard, and who are effectively informed of their exposure and risk factors, are better equipped to be engaged citizens at Superfund sites.

## Figures and Tables

**Table 1:** Maximum elemental concentration (µg/m^3^) measured using ICP-MS and collected during the PM_100_ monitoring period. Values in parenthesis are percent detected. “Greeley” and “Greeley duplicate” are the same location monitored twice for quality control. Two sites (A & B) were non-detect (ND) for all elements.

	Walkerville (A)	Excelsior (B)	Colorado St. (C)	Greeley (D)	Greeley Duplicate (D)	Earth Ln. (E)
Lead	ND	ND	ND	ND	ND	ND
Molybdenum	ND	ND	3.5 (33%)	4.5 (17%)	14.4 (33%)	0.0
Copper	ND	ND	10.0 (17%)	34.7 (33%)	48.1 (50%)	9.6 (17%)
Manganese	ND	ND	ND	ND	ND	ND
Zinc	ND	ND	50.2 (33%)	33.0 (17%)	ND	ND
Arsenic	ND	ND	ND	ND	ND	ND

**Table 2:** Categories characterizing aggregated (pre & post) participant qualitative responses. Numbers represent total aggregated comments—positive and negative—associated with each category.

	Water	Soil	Air
a. Location in town: uptown/flats; proximity to active mine/historic mining wastes	10	18	8
c. Familiarity with/trust for remedial programs, policies, & science	16	12	8
e. Appearance, taste, smell, texture (e.g., soil type)	10	24	9
f. Prior experience/information (including misinformation)	11	8	13
g. Uncertainties/worries about contaminants; how affected by screening	7	7	7
h. Screening results validate original/existing perceptions	20	18	19

## Data Availability

The data presented in this study are available on request from the corresponding author.
